# Dynamic Analysis of the Fruit Sugar-Acid Profile in a Fresh-Sweet Mutant and Wild Type in ‘Shatangju’ (*Citrus reticulata* cv.)

**DOI:** 10.3390/plants13192722

**Published:** 2024-09-28

**Authors:** Xiangyang Li, Yuan Zeng, Ting Wang, Bo Jiang, Mingjing Liao, Yuanda Lv, Juan Li, Yun Zhong

**Affiliations:** 1 Institute of Fruit Tree Research, Guangdong Academy of Agricultural Sciences, Guangzhou 510640, China; lixiangyang@gdaas.cn (X.L.); jiangbo10086@126.com (B.J.); 2 Key Laboratory of South Subtropical Fruit Tree Biology and Genetic Resources Utilization, Ministry of Agriculture and Rural Affairs/Guangdong Provincial Key Laboratory of Science and Technology Research on Fruit Tree, Guangzhou 510640, China; yuanzeng0317@163.com (Y.Z.); wangtingfafu@126.com (T.W.); l759819241@163.com (M.L.); lvyuanda2008@163.com (Y.L.); 3 College of Horticulture, Zhongkai University of Agriculture and Engineering, Guangzhou 510225, China

**Keywords:** *citrus*, high-performance liquid chromatography (HPLC), metabolic profile, organic acids, sugars

## Abstract

Citrate is a major determinant of fruit flavor quality. Currently, *citrus* species and/or varieties with significant alterations in citrate level have greatly advanced the molecular basis of citrate accumulation in fruit. However, in-depth dissections of the molecular mechanism specific to citrate accumulation are still limited due to the lack of mutants, especially within one single variety. In this study, a fresh-sweet ‘Shatangju’ mutant (*Citrus reticulata* cv.) was obtained during a survey of *citrus* resources in Guangdong, China, and the phenotype, fruit morphology, and primary flavor profiles were comparatively analyzed. Unlike the wild-type ‘Shatangju’ (WT), the mutant (MT) material exhibited a dwarfed and multi-branched tree shape, delayed flowering and fruit ripening at maturity, a prolonged fruit tree-retention time, and a decreased single fruit weight at maturity. Dynamic measurement of the metabolite levels further suggested that the contents and fluctuation patterns of vitamin C, malate, quinate, and oxalate showed no obvious difference between MT and MT fruits, while the citrate level in MT fruits significantly decreased over various developmental stages, ranging from 0.356 to 1.91 mg g^−1^ FW. In addition, the accumulation patterns of the major soluble sugars (sucrose, fructose, and glucose), as well as the sugar/acid ratio, were also altered in MT fruits during development. Taken together, this study provides a novel acid-free ‘Shatangju’ mutant, which can serve as a powerful tool for the research of fruit flavor quality, especially for the comprehensive understanding of the molecular mechanism of citrate accumulation in fruits.

## 1. Introduction

Citrus (Rutaceae, Sapindales) is an important fleshy fruit for human health, and plays a vital role in the agricultural economy and food industry worldwide. Due to long-term intraspecific and interspecific hybridization, as well as clonal propagation, the modern cultivars comprise a wide variety of *citrus* species, such as mandarin (*Citrus reticulata*), sweet orange (*Citrus sinensis*), pummelo (*Citrus grandis*), lemon (*Citrus limon*), and grapefruit (*Citrus paradise*). All these species together result in diverse fruit quality attributes, encompassing fruit size, aroma profile, sweetness, and acidity, as well as vitamin and flavonoid composition. The combination of these traits collectively determines the *citrus* flavor and nutritional value, which directly impacts customer acceptance and economic value [1,2].

As the major determinants of fruit taste and flavor quality in fruit crops, organic acids, mainly citrate and malate, can affect the perception of sweetness, and the ratio of sugars to organic acids is a key attribute influencing the harvest time of fleshy fruit and consumers’ acceptance [3]. Moreover, organic acids function importantly in multiple physiological processes, such as plant growth, stress tolerance, and pH homeostasis [4,5,6]. In citrus, excessively high or low levels of citrate greatly impair the fruit palatability, and manipulating fruit sweetness by altering organic acid concentrations has increasingly become a priority for improving fruit quality [7]. For example, in high-acid *citrus* varieties such as sour lemon and lime, the levels of citric acid reach up to 60 mg mL^−1^, while it does not exceed 1 mg mL^−1^ in acidless cultivars [6]. Therefore, improving the abundance of *citrus* genetic materials with diversity in citrate contents is an important fundament for the dissection of the molecular mechanisms underlying citrate accumulation in *citrus* fruits, which will ultimately benefit molecular design breeding and selection of varieties with varying acid levels.

The citrate level in fleshy fruit is determined by the balance of its biosynthesis, degradation, and vacuolar storage [5,6,8]. Currently, the synthesis and degradation pathways of citrate have been extensively studied, and the physiological functions and regulatory modes of key structural genes have also been widely reported [9]. Citrate metabolism typically involves four pathways, i.e., the tricarboxylic acid cycle in the mitochondrion, the glyoxylate cycle in the glyoxysome, dicarboxylate conversion of citrate, and decarboxylation of malate and oxaloacetate in the cytosol [3]. In contrast, it is believed that the accumulation levels of citrate in fleshy fruits are largely determined by the transportation from the cytosol to the vacuole, which plays a key role in storing excessively high levels of citrate. So far, great advances have been made concerning the molecular basis of the regulation of citrate accumulation in *citrus* fruits, based on abundant genetic material and multi-omics tools. For instance, CsCit1, a vacuolar citrate/H^+^ symporter, might induce efflux of citrate from the vacuole when *citrus* fruit ripens. In ‘Ponkan’ fruit, overexpressing or silencing of a negatively correlated transporter CitALMT (aluminum-activated malate transporter) significantly alters the citrate content [10]. Importantly, studies on multiple *citrus* species including lemon, mandarin, pummelo, and orange, have suggested that P-type proton pumps CitPH1 and CitPH5, localized on the vacuolar membrane, form a complex to transport the influx of protons (H^+^) from the cytoplasm into the vacuole lumen, thus promoting vacuole hyper-acidification and providing a driving force for citrate transport in *citrus* fruits [2,7]. In addition, a number of transcription factors related to citrate accumulation have been identified. In ‘Ponkan’ fruit, CitERF6 binds and activates the *CitACLα1* promoter to reduce citric acid content, while CitERF13 promotes citrate accumulation by interacting with the proton pump subunit CitVHA-c4 [8,11]. Notably, through a comprehensive genetic analysis of various *citrus* species with different citrate levels, a hierarchical regulatory cascade CitPH4-CitAN1-CitTRL has been dissected that modulates citrate accumulation via regulating the expression of proton pump CitPH1 and CitPH5. Specifically, the R2R3 MYB CitPH4 is capable of binding and activating the promoters of CitAN1 (Noemi), CitTRL, and CitPH5; CitPH4 interacts with CitAN1 (a bHLH protein) to form the CitPH4-CitAN1 complex, thus strongly inducing the expression of *CitPH5* and promoting vacuolar hyper-acidification, while R3 MYB CitTRL competes with CitPH4 for binding to CitAN1, thus down-regulating *CitPH5* expression and inhibiting citrate accumulation [2,12,13,14]. Identification and further functional dissection of the proton pumps and citrate transport-related transporters on tonoplasts have been the focus in recent years. However, much remains to be explored regarding the molecular mechanisms specific to citrate accumulation in *citrus*, and the abundance of genetic material needs to be further improved.

Citrus is a major source of citrate, which accounts for 90% of the organic acids that accumulate in fruit [15]. Although its biosynthesis has been illustrated, regulatory mechanisms of citrate accumulation remain to be dissected. Therefore, a large number of *citrus* species with significant alterations in their citrate levels have been employed for studies on plant citrate metabolism, such as kumquat (*Citrus crassifolia*), lemon, pummelo, mandarin, and sweet orange [6]. However, mutants with extremely low citrate content within one single variety are scarce. In this study, a fresh-sweet mutant (MT) ‘Shatangju’ (*Citrus reticulata* cv.) was obtained during a survey of *citrus* resources in Guangdong, China, and morphology of the MT tree, especially the fruit morphology in various developmental stages, were first investigated. Further, the major fruit quality indicators (organic acids and soluble sugars) of MT and wild-type (WT) ‘Shatangju’ fruits, as well as the sugar/acid ratios, were comparatively analyzed over various developmental stages, via high-performance liquid chromatography (HPLC)-based metabolite detection. Based on the above investigations, a mutant with extremely low citrate levels was presented, which will greatly facilitate the research on the molecular mechanism of fruit citrate accumulation in the future.

## 2. Results

### 2.1. Morphological Characterization of Tree and Fruit in Normal ‘Shatangju’ (WT) and ‘Shatangju’ Mutant (MT)

**Figure 1:**
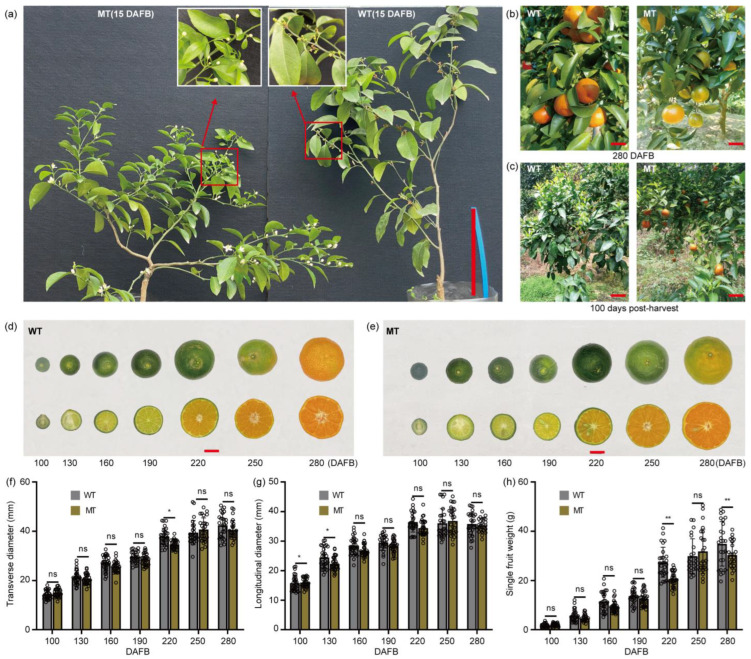
Tree form and fruit morphology of WT and MT materials. (**a**) Tree form and flowering phenotypes of greenhouse-grown WT and MT trees. The inset panels indicated by red arrow and box combination represent localized magnified images. Two consecutive years of observations (i.e., March 2023 and March 2024) with consistent results. Scale bar, 15 cm. DAFB, days after full bloom. (**b**) Fruit ripening of the orchard-grown WT and MT trees at maturity (280 DAFB, 25 December 2023). Scale bar, 3 cm. (**c**) Fruit retention on the orchard-grown WT and MT trees 100 days postharvest (i.e., March 2024). Scale bar, 9 cm. (**d**,**e**) Photographs of the WT (**d**) and MT (**e**) fruit morphology at various developmental stages (100–280 DAFB). Scale bar, 1 cm. (**f**–**h**) Quantitative indicators of fruit morphology. Transverse diameters (**f**), longitudinal diameter (**g**), and single fruit weight (**h**). Error bars indicate SD (*n* ≥ 27), * *p* < 0.05, ** *p* < 0.01, ns indicates no significance, two-tailed Student’s *t*-test.

The ‘Shatangju’ MT was originally found in a commercial orchard in Shaoguan (Guangdong, China) as a spontaneous bud mutation from the normal ‘Shatangju’. Morphological analysis showed that, compared with the WT tree, the MT tree was dwarfed and multibranched with delayed flowering time (Figure 1a). Moreover, fruits of the MT trees also exhibited delayed ripening at maturity (280 days after full bloom, DAFB) (Figure 1b,d,e) and prolonged tree-retention time that continued until the flowering period in March of the following year (Figure 1c). In addition, compared with WT fruits, the single fruit weight of the mutant was significantly decreased at maturity (280DAFB) by about 12.9% (Figure 1d–h), though the transverse diameter and longitudinal diameter showed no obvious difference during development.

### 2.2. Chromatogram of Organic Acids and Sugars

**Figure 2:**
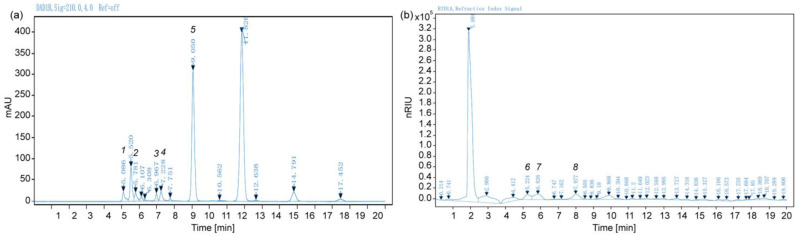
Chromatograms for organic acids (**a**) and soluble sugars (**b**). The numbers labeled in sequence from 1 to 8 represent the peak times of different metabolite standards. 1–5: oxalate, quinate, L-malate, vitamin C (ascorbic acid), citrate; 6–8: fructose, glucose, sucrose.

At the fully mature stage (280 DAFB), the MT fruits had a fresh-sweet flavor with significantly reduced acidity. As is known, sweetness and acidity, the main components of *citrus* taste and flavor quality, are primarily dominated by the type and concentration of soluble sugar and organic acid [1]. Therefore, HPLC was performed to detect the major organic acids and soluble sugars. As shown in Figure 2, standards of the major organic acids and sugars were well separated in the chromatograms. Moreover, the correlation coefficients of each metabolite were greater than 0.99, indicating a strong correlation and high reliability (Table 1).

### 2.3. Dynamic Profiles of Organic Acids during the Development of WT and MT Fruits

**Figure 3:**
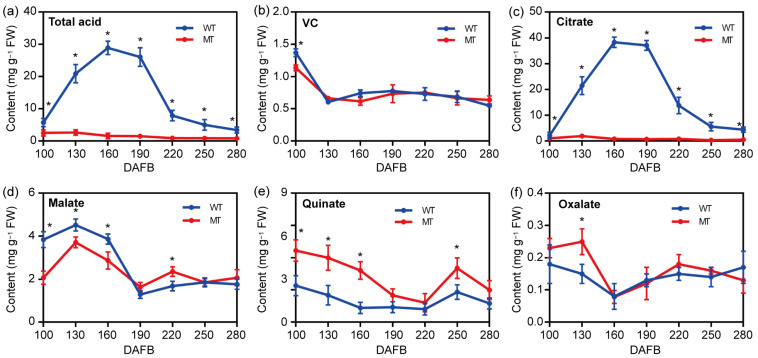
Dynamic profiles of the major organic acids in WT and MT fruits over different developmental stages. (**a**) Total acidity, i.e., titratable acidity; (**b**) VC (ascorbic acid); (**c**) citrate; (**d**) malate; (**e**) quinate; (**f**) oxalate. DAFB, days after full bloom. All experiments were repeated at least twice. Error bars indicate SD (*n* = 3 biological replicates), * *p* < 0.05, two-tailed Student’s *t*-test.

To explore the dynamics of organic acid contents during the development of WT and MT fruits, seven developmental stages of WT and MT fruits (100, 130, 160, 190, 220, 250, and 280 DAFB) were sampled. As can be seen from Figure 3a,b, the vitamin C (VC) content showed consistent dynamics between MT and MT fruits over various development stages, while total acid content in MT fruits significantly decreased. Then, the contents of the three predominate organic acid components (citrate, L-malate, and quinate), as well as oxalate, were measured. As shown in Figure 3d, the dynamics of malate content in WT and MT fruits were similar over various developmental stages, which peaked at 130 DAFB and slowly decreased to approximately 2.0 mg g^−1^ FW at 280 DAFB. Likewise, the content dynamics of both quinate and oxalate between WT and MT fruits showed roughly similar patterns during fruit development, which gradually decreased to a comparable level at maturity (280 DAFB), but with a slightly higher level of quinate in MT fruits (Figure 3e,f). In contrast, the citrate content in MT fruits was extremely low over all of the developmental stages, ranging from 0.356 to 1.91 mg g^−1^ FW. In WT fruits, the citrate content exhibited a continuous accumulation pattern from 1.83 mg g^−1^ FW at 100 DAFB to 38.3 mg g^−1^ FW at 160 DAFB, and then rapidly decreased to 4.41 mg g^−1^ FW at maturity (280 DAFB). The above results suggest that the difference in acidity of the MT fruits mainly results from the extremely low citrate content.

### 2.4. Dynamic Profiles of the Soluble Sugars during the Development of WT and MT Fruits

**Figure 4:**
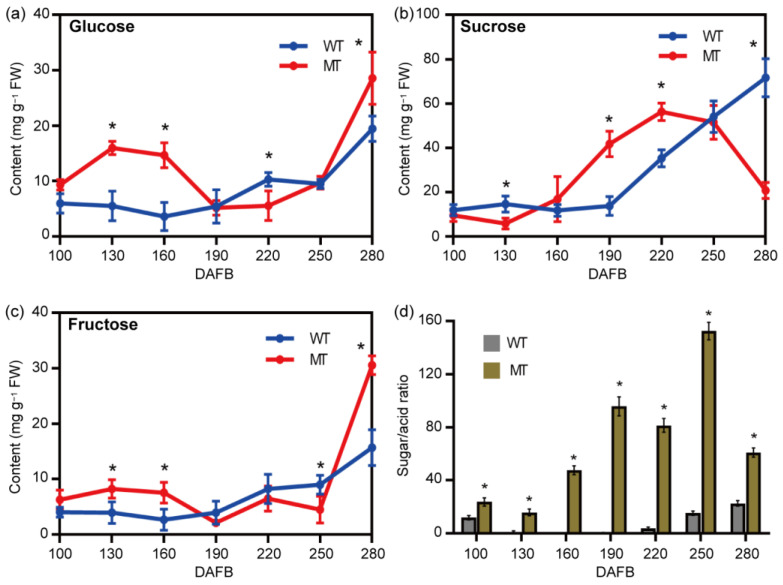
Dynamic profiles of the major soluble sugars in WT and MT fruits over different developmental stages. (**a**–**c**), Glucose, sucrose, and fructose, respectively; (**d**), sugar/acid ratio, for which, contents of the major soluble sugars (sucrose, glucose, and fructose) measured by HPLC were divided by that of citric acid to obtain the sugar/acid ratio. DAFB, days after full bloom. All experiments were repeated at least twice. Error bars indicate SD (*n* = 3 biological replicates), * *p* < 0.05, two-tailed Student’s *t*-test.

Next, measurements were made for the primary sugar content (i.e., sucrose, fructose, and glucose) in WT and MT fruits. The results showed that while the sucrose content in MT fruits remained largely unchanged, the contents of glucose and fructose increased from 100 DAFB to 160 DAFB. Subsequently, their contents gradually decreased from 160 DAFB to 250 DAFB, coinciding with a significant increase in sucrose content. Upon reaching the ripening stage (280 DAFB), the sucrose content sharply decreased, accompanied with a significant increase in the contents of glucose and fructose. In contrast, the concentrations of the three soluble sugars did not change obviously from 100 DAFB to 190 DAFB, but increased significantly from 190 DAFB to 280 DAFB (Figure 4a–c). The aforementioned results showed the altered pattern of soluble sugar accumulation in the MT fruits during various developmental stages.

The sugar/acid ratio is recognized as the major determinant of fruit sweetness and ripeness [7]. Therefore, the sugar/acid ratio was calculated. The results showed that over the different developmental stages, the sugar/acid ratios of MT fruits were significantly higher than that of the WT fruits, and increased from 15.77 to 152.58 at 100 DAFB to 250 DAFB. At maturity, the sugar/acid ratio in MT fruits decreased to 61.03, but it was still higher than that of the WT group at 22.64 (Figure 4d).

## 3. Discussion

In recent years, natural shoot mutations have become an important resource for breeding *citrus* and other fruit trees [16,17,18,19]. The mutant investigated in this study is derived from a spontaneous mutation in Guangdong ‘Shatangju’, and exhibits novel phenotypes in *citrus*, including a dwarfed and multi-branched tree shape, delayed flowering and fruit ripening at maturity, and prolonged fruit tree-retention time (Figure 1a–d). These investigations suggested that some traits related to the fruits were the main source of the notable distinctions between MT and WT materials, thus, further in-depth analysis of the MT fruits was carried out in this study. Fruit morphology is the outward manifestation of genetic traits, as well as the intuitive basis for population classification and species identification [20]. The statistics showed there were no obvious differences in the transverse diameter and longitudinal diameter of the WT and MT fruits over various developmental stages, but the single fruit weight in MT significantly decreased at maturity (Figure 1f–h). It has been reported that fruit quantity traits are closely related to the nutrient supply and the age of the fruit tree [21,22]. Therefore, the decreased single fruit weight in MT may result from metabolite competition between vegetative and reproductive growth.

Next, the major flavor quality of this MT fruit was analyzed via HPLC-based measurements. The results showed that the contents of VC, malate, quinate, and oxalate had no obvious difference between WT and MT fruits over various development stages (Figure 3b,d–f). However, the content dynamics of citrate between WT and MT fruits showed a great difference. Specifically, the citrate content of WT fruits exhibited a continuous accumulation pattern from 1.83 mg g^−1^ FW to 38.3 mg g^−1^ FW, and then rapidly decreased to 4.41 mg g^−1^ FW at maturity (280 DAFB). In contrast, the citrate content in MT fruits was extremely low over all of the developmental stages, ranging from 0.356 to 1.91 mg g^−1^ FW (Figure 3c). In the *citrus* kingdom, the fluctuations of citrate accumulation follow a “rising and then falling” pattern, which peaked at 150 DAFB and declined thereafter [23]. Consistently, the citrate content measured in WT fruits initially increased at 100 DAFB, peaked at 160 DAFB, and then declined to the lowest level at 280 DAFB (Figure 3a,c). Moreover, changes in the content of titratable acids can indirectly reflect the alteration of citrate levels in fruits, which accounts for >90% of the organic acids in *citrus* fruits [15]. In this study, the fluctuation patterns of total acid levels were similar to that of the citrate levels in WT and MT fruits (Figure 3a), which further validates the reliability of the measured data. The citrate contents in different *citrus* varieties vary dramatically based on which *citrus* types are classified into different acidity varieties, i.e., extremely high-acid types (>15 mg g^−1^ FW), high-acid types (10–15 mg g^−1^ FW), moderate-acid types (6–10 mg g^−1^ FW), low-acid types (2–6 mg g^−1^ FW), and non-acidic types (<2 mg g^−1^ FW) [24,25]. Thus, the extremely low level of citrate in MT fruits indicates the taxonomic nonacidic nature of this variety.

In addition, the major soluble sugars (sucrose, fructose, and glucose) were also measured in this study. The sucrose content in MT fruits remained largely unchanged, while the contents of glucose and fructose increased from 100 DAFB to 160 DAFB, which subsequently decreased from 160 DAFB to 250 DAFB, coinciding with a significant increase in the sucrose content. However, at maturity (280 DAFB), the sucrose content sharply decreased, accompanied with a significant increase in the contents of glucose and fructose. In contrast, the contents of the three soluble sugars in WT fruits did not change obviously from 100 DAFB to 190 DAFB, but increased significantly from 190 DAFB to 280 DAFB (Figure 4a–c), which is in line with the dynamic patterns of concurrent rise and fall in the concentrations of sugars during fruit ripening [26,27,28]. Therefore, these results suggested the accumulation patterns of the major soluble sugars were altered in MT fruits.

The flavor profile resulting from sweet and sour fruits is mainly determined by the interplay between the type and proportion of sugar and acid contents and their ratio [3,29,30]. Fruits with a sugar/acid ratio below 14.9 tend to be predominantly sweet-sour or sour, while those with a ratio ranging from 15 to 25 are mostly sweet-sour. A sugar/acid ratio ranging from 25.1 to 60 typically results in a sweet and sour flavor profile. Conversely, when the sugar/acid ratio exceeds 60.1, the flavor profile is generally characterized as light, sweet, or highly sweet [31,32]. In this study, the sugar/acid ratio in WT fruits was 22.64, suggesting the flavor profile is mainly sweet-sour. In comparison, the MT fruits predominantly exhibited a fresh-sweet flavor, as evidenced by the high sugar/acid ratio (61.03) in the fruits.

Currently, most *citrus* varieties contain some amount of citrate in their ripe fruits, while the acid-free varieties are relatively rare [33]. In this study, an intraspecific ‘Shatangju’ mutant with extremely low citrate was identified through the integrated evaluations of the phenotype, fruit morphology, and the major sugar-acid profiles, which will serve as an acid-free genetic material to further enrich the abundance of acid-free *citrus* varieties and benefit molecular breeding for flavor regulation.

## 4. Materials and Methods

### 4.1. Experimental Materials and Measurements

In this study, the wild-type (WT) ‘Shatangju’ (*Citrus reticulata* cv.) and its mutant (MT), sourced from a commercial orchard in Xinfeng County, Shaoguan City, Guangdong Province, China, were employed for morphological observations and various quantitative determinations. The geographic coordinates of the commercial orchard are located at latitude 23.994094° N and longitude 113.909814° E, and the climate in Xinfeng County is monsoon sub-tropic with an annual precipitation of 1911.8 mm, average annual temperature of 20.5 °C, and average annual sunlight of 1467 h (https://www.xinfeng.gov.cn/; accessed on 19 September 2024). Additionally, the MT and WT trees, grafted for more than 2 years (i.e., from December 2021 to March 2024) in a greenhouse facility (operated by the Fruit Tree Research Institute of the Guangdong Academy of Agricultural Sciences), were also used for some of the analyses in this research.

For morphological observations, the MT and WT materials were photographed with the greenhouse-grown trees or the orchard-grown trees for their flowering times and tree-retention times of the fruit. To dynamically analyze the morphological indicators of MT and WT fruits at various developmental stages, including transverse diameter (mm), longitudinal diameter (mm), and single fruit weight (g), approximately 30 fruits per month were collected from three orchard-grown WT and MT trees for photography and statistical analysis.

For the determination of metabolite contents, eighteen fruits with similar sizes and no physical injuries were sampled from three orchard-grown MT and WT trees. Then, the harvested MT and WT fruits were peeled and divided into three groups. Each group of the MT and WT pulps was considered to be one biological replicate. Finally, the MT and WT pulps were rapidly frozen in liquid nitrogen, ground into powder, and stored at −80 °C for further use.

For all of the dynamic analyses above, fruit sampling commenced 100 days after full bloom (DAFB) (i.e., 25 June 2022), and then continued monthly until maturity (280 DAFB, 25 December 2022).

### 4.2. Chemicals and Reagents

High-performance liquid chromatography (HPLC)-grade acetonitrile was purchased from Merck (Darmstadt, Germany), and pure water was prepared with Milli-Q water (Millipore Corp., Saint-Quentin, France). Standards of sucrose, glucose, fructose, VC (ascorbic acid), quinate, malate, citrate, and oxalate, all with a purity exceeding 99.0%, were purchased from Sigma-Aldrich (St. Louis, MO, USA). H_3_PO_4_ and (NH4)_2_HPO_4_ of analytical grade were purchased from Zhiyuan Chemical Reagent Co., Ltd. (Tianjin, China) and Kemiou Chemical Reagent Co., Ltd. (Tianjin, China), respectively. All solutions used were filtrated through a 0.45 μm pore-size membrane filter (Millipore).

Standards with seven concentrations in a range of 0.0625–4.0 mg mL^−1^ for the major sugars (glucose, fructose, and sucrose), 0.01–16.0 mg mL^−1^ for the major organic acids (citrate and L-malate), and 0.03125–2.0 mg mL^−1^ for the trace organic acids (quinate, vitamin C, and oxalate) were used to create a standard curve for calculating the content of primary organic acids and soluble sugars in fruit pulp. The coefficients of variation were generally less than 7.0%.

### 4.3. Sample Extraction

Organic acids and soluble sugars were extracted according to the method described by Li, et al. [34]. Briefly, 1 g of the powdered samples was mixed with ultrapure water at a 1:5 (*w*/*v*) ratio. Then, the samples were extracted sequentially by water bath at 70 °C for 15 min, ultrasonication for 15 min, and centrifugation for 15 min. After that, the collected supernatant was filtered through a 0.22 μm filter and used for subsequent testing.

### 4.4. High-Performance Liquid Chromatography (HPLC) Analysis of Sugars and Organic Acids

The contents of major organic acids and sugars in MT and WT fruit pulp were measured according to the method described by Li, et al. [34], by using high-performance liquid chromatography (HPLC) (Waters Corp., Wilford, MA, USA). Each experiment was carried out with three biological replicates.

A diode array detector (DAD) was used to detect organic acids at 210 nm. The C18 chromatographic column (Agilent ZORBAX SB-Aq, Santa Clara, CA, USA) was used with a column temperature of 37 °C. The mobile phase was composed of potassium dihydrogen phosphate (6 mM, pH 2.5), methanol, and distilled water. All mobile phases were filtered through a 0.45 μm membrane and degassed by ultrasonication before machining. The determination duration was 20 min, and the flow rate was fixed at 0.6 mL min^−1^.

Soluble sugars were detected using a refractive index detector (RID). An amino acid column (Agilent Carbohydrate) was used with a column temperature of 35 °C, and the mobile phase consisted of acetonitrile and ultrapure water in a 0.75:0.25 (*v*/*v*) ratio. All mobile phases were filtered through a 0.45 μm membrane and then degassed by ultrasonication before machining. The determination duration was 20 min, and the flow rate was fixed at 0.6 mL min^−1^.

### 4.5. Determination of Titratable Acidity Contents

A volume of 5 mL juice from the WT and MT groups was diluted in 20 mL distilled water and then titrated with 0.1 M NaOH to the endpoint at pH 8.2, according to a previous report [35], using a citric acid coefficient of 0.064. Titratable acidity (TA) was calculated as a percentage of citric acid.

### 4.6. Statistical Analysis

Data analyses were conducted with GraphPad Prism 8 software (Version 8). Differences of the quantitative indicators between MT and WT groups were assessed using a two-tailed Student’s *t*-test. The results were presented as mean ± SD. * *p* < 0.05 and ** *p* < 0.01 were considered to be statistically significant differences, with ns indicating no significance. Graphs were obtained using Adobe Illustrator 2021.

## 5. Conclusions

This is the first investigation of the acid and sugar alterations associated with a fresh-sweet fruit mutation in ‘Shatangju’. In this study, the phenotype, fruit morphology, and the major flavor profiles (organic acid and soluble sugar) were comparatively analyzed for the ‘Shatangju’ MT and its WT. Compared with the morphological traits of the ‘Shatangju’ WT, the MT material exhibited delayed flowering time and fruit ripening at maturity, and prolonged fruit retention time on the tree, as well as decreased single fruit weight at maturity. HPLC-based dynamic measurements of the major organic acids and soluble sugars further showed that, during various developmental stages, the contents of VC, malate, quinate, and oxalate had no obvious difference between MT and MT fruits, while the citrate content in MT fruits significantly decreased, ranging from 0.356 to 1.91 mg g^−1^ FW. Moreover, the accumulation patterns of the major soluble sugars (sucrose, fructose, and glucose), as well as the sugar/acid ratio, were altered in MT fruits during development. Overall, investigations in this study characterized a valuable breeding material, i.e., the acid-free ‘Shatangju’ mutant, which will shed new light in the future to facilitate an in-depth understanding of the molecular regulation of citrate accumulation in *citrus* fruits.

## Figures and Tables

**Table 1:** Analytical characteristics of the selected organic acid and soluble sugar standards by HPLC.

Compound	Peak Time (min)	Linear Equation (Integrator Response to Concentration (mg mL^−1^), *n* = 3)	Correlation Coefficient (*R*^2^)
Glucose	5.826	y = 5 × 10^−6^ x + 0.0046	0.999
Fructose	5.224	y = 6 ×10^−6^ x − 0.0433	0.997
Sucrose	7.977	y = 6 × 10^−6^ x − 0.0691	0.992
Citrate	9.050	y = 8 × 10^−4^ x + 0.0096	0.9999
L-Malate	6.967	y = 1 × 10^−3^ x + 0.0001	1.000
Quinate	5.781	y = 1.5 × 10^−3^ x − 0.0151	0.9994
Vitamin C	7.228	y = 9 × 10^−5^ x + 0.1075	0.997
Oxalate	5.086	y = 5 × 10^−5^ x − 0.0087	0.9995

## Data Availability

Data are contained within the article.
